# Transforming Primary Care Practice Into Age-Friendly Health Systems

**DOI:** 10.1093/geroni/igaa057.2588

**Published:** 2020-12-16

**Authors:** Marla Berg-Weger, Erin Emery-Tiburcio, Nina Tumosa

## Abstract

Transforming primary care practice to improve the health of older adults is a major focus of the Geriatric Workforce Enhancement Program (GWEP). Using the 4Ms of an Age-Friendly Health System (What Matters, Mentation, Medication, and Mobility) as a framework, presenters will describe their GWEP’s ongoing development, training, and evaluation initiatives aimed at increasing providers’ knowledge and practice skills, and improving older adult’s health outcomes. These initiatives are creating increased professional competencies in geriatric care that will: 1) help older adults maximize their health and wellbeing, and 2) better support caregivers and families. In this symposium, presenters from three GWEPs, Pennsylvania State University, Rush University Medical Center, and Saint Louis University will describe AFHS initiatives with rural and urban primary care partner sites. Educational and programmatic initiatives and strategies that map onto the 4Ms that will be discussed including geriatric assessment, dementia-focused interventions, falls prevention, opioid assessment, and caregiver well-being and support. Outcomes in older adult’s health and functional status will be discussed. Presenters will highlight the importance of building AFHS in primary care and strategies to bridge this framework into the community.

